# Receptor Activator of Nuclear Factor Kappa B (RANK) and Clinicopathological Variables in Endometrial Cancer: A Study at Protein and Gene Level

**DOI:** 10.3390/ijms19071848

**Published:** 2018-06-22

**Authors:** Raúl Gómez, Ana Castro, Jessica Martínez, Víctor Rodríguez-García, Octavio Burgués, Juan J. Tarín, Antonio Cano

**Affiliations:** 1 Research Unit on Women’s Health—Institute of Health Research INCLIVA, 46010 Valencia, Spain; raulgomgal@gmail.com (R.G.); jmartinez@incliva.es (J.M.); victor.rodriguez-garcia@uv.es (V.R.-G.); 2 Department of Pediatrics, Obstetrics and Gynecology, University of Valencia, 46010 Valencia, Spain; anacastroperez1986@gmail.com; 3 Pathology Department, Hospital Clinico Universitario, 46010 Valencia, Spain; octavio.burgues@uv.es; 4 Department of Cellular Biology, Functional Biology and Physical Anthropology, University of Valencia, 46100 Burjassot, Spain; Juan.J.Tarin@uv.es

**Keywords:** RANK, endometrium, endometrial cancer, prognosis, immunohistochemistry, gene expression

## Abstract

The system integrated by the receptor activator of nuclear factor kappa B (RANK) and its ligand, RANKL, modulates the role of hormones in the genesis and progression of breast tumors. We investigated whether the expression of RANK was related with clinicopathological features of primary endometrial tumors. Immunohistochemistry was used in an endometrial cancer tissue array containing samples from 36 tumors. The amount of RANK mRNA was examined in a tissue scan cDNA array containing cDNA from 40 tumors. Normal endometrium was examined for comparison. Immunohistochemical analyses showed that RANK expression was higher in malignant than in normal endometrium (*p* < 0.05). RANK expression was related to histological grade (Pearson correlation index = 0.484, *p* < 0.001), but not to tumor stage or to age of the women. The gene expression was similar in malignant and normal endometrium. The study of RANK isoforms confirmed that the overall relative abundance of the three clearly identified transcripts was similar in normal and pathological endometrium. RANK protein expression increased from normal to malignant endometrium, and the expression level was related with tumor grade but not with stage or the age of subjects in endometrial cancer. In contrast, similar comparisons showed no change in RANK gene expression.

## 1. Background

Endometrial tumors are members of the big family of endocrine-related cancer. This is certified by their frequent expression of estrogen and progesterone receptors and by their hormonal sensitivity [1]. Moreover, the long-term exposure to estrogens is a risk factor whose impact is reduced by progestogens [2,3]. Tumors from other reproductive-related organs, like the breast, are also endocrine sensitive.

Studies published in the latter years have shown that the system integrated by the receptor activator of nuclear factor kappa B (RANK) and its ligand, RANKL, may have a role in breast cancer oncogenesis (reviewed in [4]). RANK and RANKL are members of the tumor necrosis factor (TNF) family of cytokines [5]. Activation of members from the TNF family has a multifunctional role, including inflammation, organogenesis, apoptosis, or immunological functions [6]. Of interest, malignant breast cancer cells express RANK and RANKL, and an association between the expression of RANK and parameters of aggressiveness of breast tumors has been found in experimental studies [4]. Furthermore, recent data suggest a role for RANKL/RANK in unstable mammary cell populations of BRCA1 mutation carriers (already bearing a molecular signature similar to that of basal breast cancer) [7]. Parallel clinical data have found that RANK expression is related with some important prognostic parameters, like survival or metastatic potential [8,9].

The data on RANK/RANKL in breast cancer raises questions about a potential role in endometrial cancer. Recent work has confirmed that RANKL/RANK are expressed in human endometrium and that the expression is increased in endometrial cancer. The immunohistochemical (IHC) levels of RANK seemed to relate with patients’ survival or metastatic status of the tumor, but not with prognostic clinicopathological parameters, such as tumor grade of differentiation [10,11]. To clarify that apparent paradox, we have designed a comprehensive methodological approach in which both IHC and quantitative gene expression were used to investigate RANK in primary endometrial tumors from which clinicopathological features were available. In a more particular analysis, we explored whether an association exists with gene expression of specific RANK isoforms.

## 2. Results

### 2.1. Immunohistochemical Analyses

**Figure 1:**
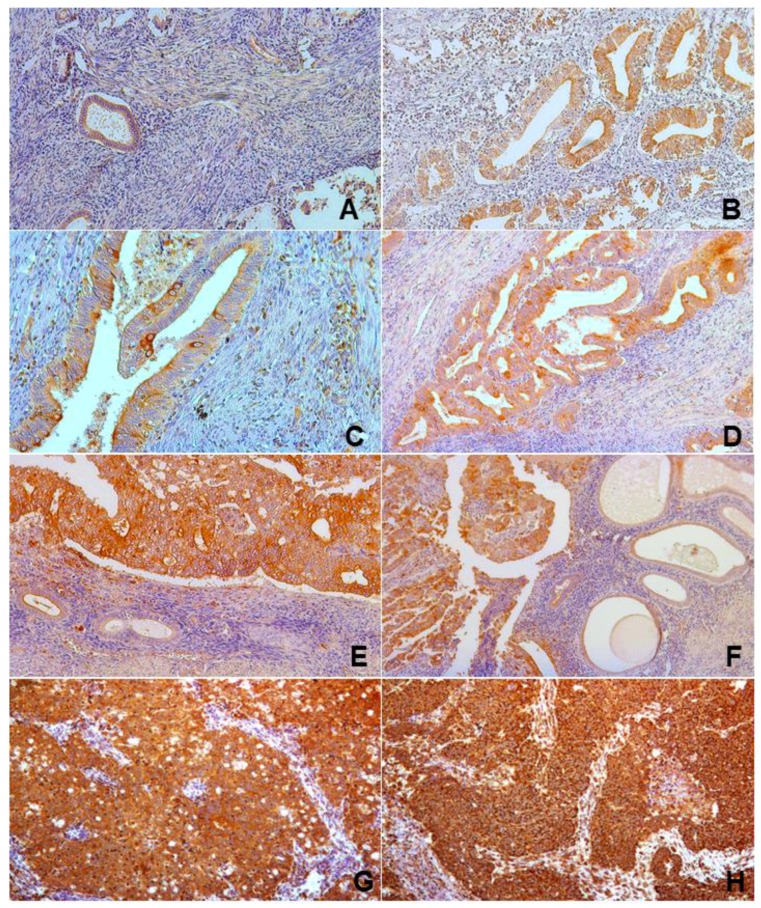
Representative patterns of RANK immunohistochemical staining (**brown** color) in normal (**A**), hyperplastic (**B**), and malignant (**C**–**H**) endometrium. Illustrative examples of the histological grade I (**C**,**D**), II (**E**,**F**), and III (**G**,**H**) tumor tissues are shown. Intense staining in epithelium of all samples with widely increased signal in undifferentiated areas of malignant tissues (**C**–**H**) was observed. Signal augmented gradually with the increasing histological grade. Note clear cytoplasmic as well as nuclear staining in epithelial and stroma cells of malignant tissue (**C**). A typical honeycomb staining pattern for RANK was observed in malignant tissue of different histological grade tumors (**D**,**E**). Note increased signaling in RANK staining in the upper (malignant) vs. lower (normal) areas of the tissue in (**E**). Also, note wider distribution of RANK signaling in histological grade III tumors (**G,H**) vs. lower grade (**C**–**F**) and normal (**A**) endometrium. Magnifications, ×100 (**A**,**B**,**D**–**H**) and ×200 (**C**).

RANK-specific staining was widely observed in most endometrial samples, but higher intensity was found in malignant areas. Faint positive staining extended across luminal and glandular epithelium and stroma in benign endometrium. Positivity clearly accumulated at the cellular membrane, showing a typical honeycomb pattern, but cytoplasmic staining was common, particularly in malignant areas. Staining intensity was higher in epithelium than in stroma. Illustrative images of endometrial samples from healthy and malignant tissue representing the mentioned pattern of expression are shown in Figure 1.

**Figure 2:**
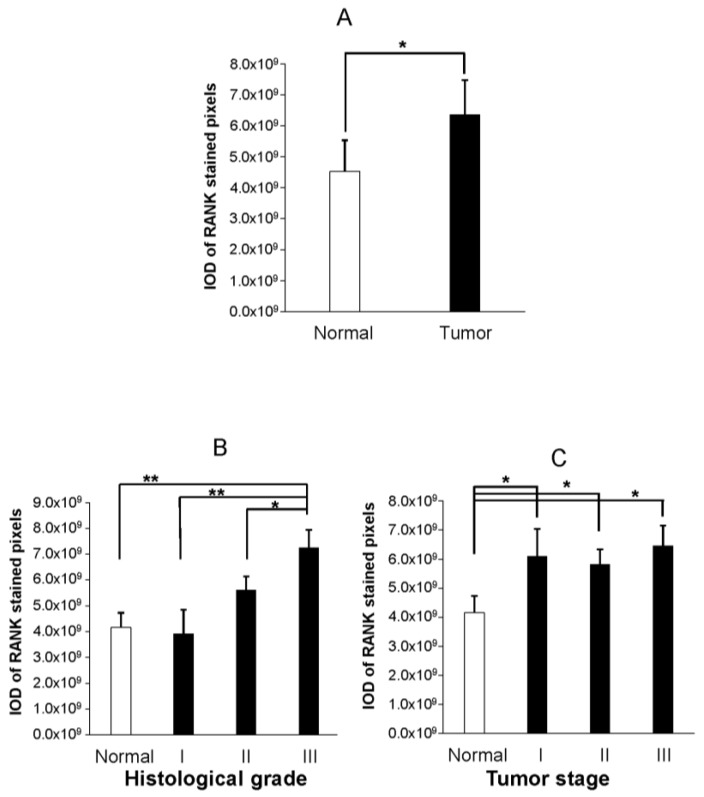
Graphs show mean ± SEM RANK protein expression as detected by quantitative immunohistochemistry in endometrium from normal and endometrial cancer samples. A Student’s *t*-test was employed for comparisons of RANK IHC staining between normal and tumor endometrial samples (**A**). An ANOVA followed by LSD post hoc analysis was performed to detect mean differences between normal and tumor samples grouped according to their histological grade (**B**) or tumor stage (**C**). Asterisks in (**B**) denote significant differences between histological grade III and the remaining (lower histological grade and normal endometrium samples) groups. Higher but not statistically significant RANK values were noted in histological grade II when compared to histological grade I or to normal samples (*p* = 0.105, *p* = 0.094, respectively). Asterisks in (**C**) denote statistically significant differences between normal and each of the groups resulting from grouping pathological samples according to their tumor stage. No differences in IHC staining values were detected when tumor stages groups were compared to each other. * *p* < 0.05, ** *p* < 0.01.

Quantitative analysis of IHC revealed that staining intensity of RANK was higher in malignant than in healthy tissue (Figure 2A, *p* < 0.05,) and correlated (Pearson) with histological grade (r = 0.484, *p* < 0.001). In separate comparisons, values of RANK staining in grade III tumor samples were significantly higher than in grade II (*p* < 0.05) or grade I (*p* < 0.01, Figure 2B) tumors. Differences in RANK expression between histological grade II and grade I tumors were of borderline significance (*p* = 0.094).

Contrary to tumor grade, we did not detect any significant correlation between tumor stage and protein RANK values (r = 0.246, *p* = 0.103). Indeed, the expression of RANK did not seem to be discriminative of tumor stage because the average RANK values were similar between stage groups (Figure 2C).

No correlation was detected between the expression of IHC RANK and age (r = 0.171, *p* = 0.327, Figure S1A). The result did not change when tumors were grouped by age range (10-year intervals, r = 0.220, *p* = 0.205, Figure S1B) or when analyses were performed within sets of tumors that were grouped by histological grade or by tumor stage.

### 2.2. PCR Analyses

**Figure 3:**
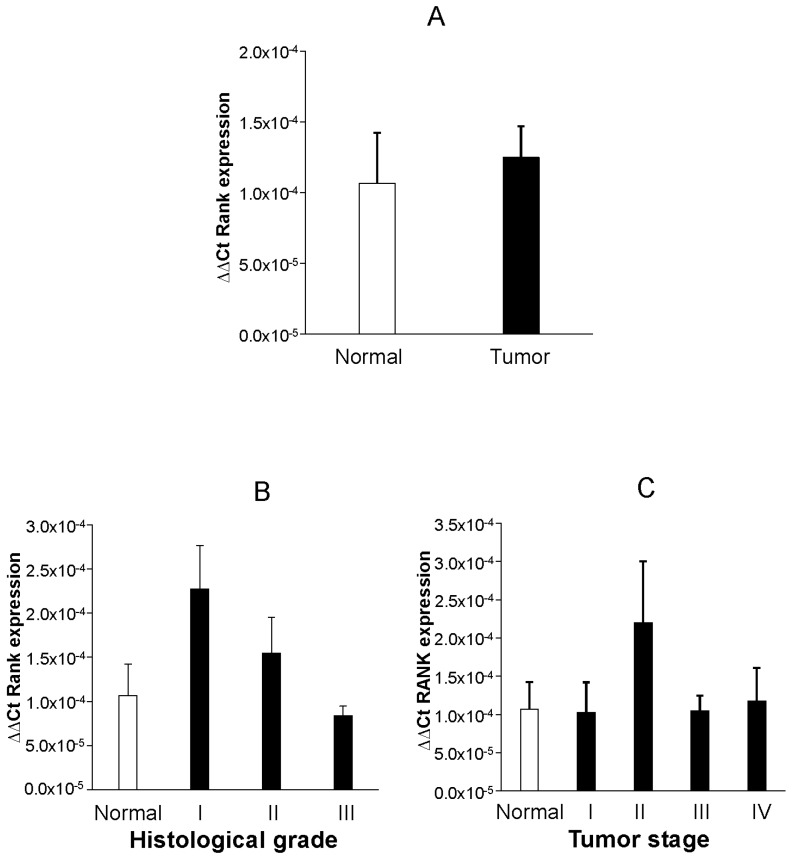
Graphs show mean ± SEM RANK mRNA expression as detected by RT-QF-PCR in endometrium from normal and endometrial tumor cases. A Mann–Whitney *U* test was employed for comparisons of RANK mRNA values between normal and overall endometrial tumor cases. (**A**) A Kruskal–Wallis followed by Mann–Whitney analysis were performed to detect differences between normal and tumor cases grouped according to their histological grade (**B**) or tumor stage (**C**). No statistically significant differences were detected.

RANK mRNA expression was consistently detected in all but one control and five tumor endometrial samples. When normalized to β-actin, relative values of RANK mRNA expression were not different between normal and malignant endometrium (Figure 3A). Analyses restricted to malignant samples could not detect significant correlation (Spearman’s Rho, r_s_) between mRNA RANK expression and histological grade (r_s_ = 0.117, *p* = 0.461), tumor stage (r_s_ = 0.098, *p* = 0.439), age (r_s_ = 0.190, *p* = 0.274, Figure S2A), or stratified age (r_s_ = 0.229, *p* = 0.187, Figure S2B). Similarly, no significant differences in mRNA RANK expression were found between groups when samples were stratified attending to histological grade (Figure 3B), tumor stage (Figure 3C), or age. Additionally, we observed that there were no differences in QF-PCR RANK mRNA when tumor cases were grouped according to the presence/absence of affected nodes (Figure S3A) or the confirmed existence of metastases (Figure S3B).

Amplification of RANK isoforms revealed different levels of expression as follows: *TNFRSF11A* > *TNFRSF11A_Δ8,9* > *TNFRSF11A_Δ7,8,9* > *TNFRSF11A_Δ9*. The *TNFRSF11A* was the highest expressed isoform, detected at Ct 28–31 and showing a 3–5-fold higher expression than *TNFRSF11A_Δ8,9*. Also, *TNFRSF11A_Δ7,8,9* showed 5–7-fold lower values than *TNFRSF11A* and thus was detected above a valid limit of detection in 50% of the cases. Finally, *TNFRSF11A_Δ9,* the lowest expressed isoform, was only detected in 6 samples and with *C*t values ranging from 35 to 40, a limitation that made us discard them for subsequent analysis. The relative abundance of the other two detected transcripts, *TNFRSF11A_Δ8,9* and *TNFRSF11A_Δ7,8,9*, was also similar in normal and pathological endometrium (Figure S4). Grouping the tumor samples according to histological grade or clinical stage did not affect the relative abundance of transcripts. Overall, these results suggest that RANK mRNA expression is not altered at the transcriptional level in endometrial tumors.

## 3. Discussion

Overall, our findings of a direct relationship between RANK expression and tumor histological grade partially differ from the only published study on primary tumors, and although consistent with that study, no association of RANK was found with tumor stage or with age [12]. The absence of metastases in our IHC series prevented us from investigating the relationship of RANK protein with that feature.

We have also investigated RANK expression at gene level in another set of tumors and have found that there were no differences between normal and malignant endometria. Accordingly, no relationship could be detected with clinicopathological parameters of tumors or when tumors were grouped according to the presence of nodal or distant metastases. The differential expression of RANK isoform genes did not change the conclusion. 

There is not an obvious explanation for the difference, despite the fact that IHC and RT-PCR data were obtained in different tumor series. The possibility that post-transcriptional upregulation might have an influence seems a possible option.

More advanced work in breast cancer may help to accommodate our apparently paradoxical findings. Immunohistochemical data have shown that RANK expression was associated with lower overall survival and with lower disease-free survival in breast tumors [8,9]. Another study using a publicly available microarray dataset of primary breast cancer patients found an association of RANK expression with skeletal metastases [13]. However, debate also exists in what refers classical clinicopathological markers in breast cancer. High RANK expression was associated with higher grade and cell proliferation, as well as with negative hormone receptors in one study [8], but the findings were not confirmed in another study [9]. 

Less information exists in what refers to more sophisticated molecular biology technology. The assessment of RANK mRNA levels found a preferential association with high tumor size and grade, and with negative estrogen receptors in one study [13], but another study using RT-PCR found that the RANK transcript levels were lower in tumors than in normal tissue samples, and reduced expression of RANK was associated with general and bone metastases or death because of the disease [14]. Despite the need for more clarifying work, the existing data have moved investigators to propose RANK expression as a prognostic marker in breast malignancies. Additionally, new work keeps providing data that add consistency to a key role of RANK/RANKL in the management of breast cancer [15]. Whether the value of RANK in endometrial cancer will reproduce that in breast cancer is still uncertain. Even so, the value of RANK as one useful marker in endometrial cancer deserves attention. The effort will be worthwhile because, should a role for RANKL/RANK be found in the genesis or progression of endometrial cancer, the possibility of using ad hoc anti-RANKL proteins might open a new area in prevention and treatment [6,16]. Looking again at the breast, the phase III clinical trial (D-CARE) is already examining the potential of denosumab, an anti-RANKL antibody, to increase the bone-metastasis-free survival [17].

## 4. Methods

### 4.1. Tissue Samples and Experimental Design

To investigate the prognostic value of RANK protein expression, specimens of normal and tumorous endometria were obtained through a tissue microarray (TMA) commercially available (catalogue number ab178155) from Abcam (Cambridge, UK). The TMA contained 48 cases of paired normal and malignant tissues with different histological grades and TNM stages. Tissues in the TMA had been arrayed according to the grade of malignancy and were all ready for IHC processing. Each case was represented by 2 formalin-fixed, paraffin-embedded 4 µm gross, 1.5-mm diameter spots on the TMA. The age (years) of cases by histological groups was (N, mean ± standard deviation, SD): normal (12, 46.75 ± 10.08); endometrial adenocarcinoma (36, 51.64 ± 8.59). The postmenopausal status of the cases was unknown. Clinicopathological features of the endometrial tumors are detailed in Table 1.

To investigate whether the amount of RANK mRNA was related to prognostic factors of endometrial cancer, a commercially available (catalogue number EDRT305) TissueScan^TM^ cDNA array (TcDA) plate, containing cDNA from endometrial tumors at different histological grades, clinical and TNM stages, was purchased from OriGene (Rockville, MD, USA). The endometrial cancer array contained cDNA (N, age in years, mean ± SD) from primary endometrial adenocarcinoma (40, 62.68 ± 12.74). The postmenopausal status of the cases included in the cancer was unknown. Clinicopathological features of the tumors included in the supplemented TcDA are detailed in Table 2.

Added to the PCR array plate were 8 exogenous cDNAs from normal proliferative endometrium that was retrieved from women (aged 30–35) undergoing voluntary sterilization at our center. The Human Ethics Review Committee of INCLIVA approved on 19 December 2013 the whole protocol and a signed consent form was obtained from women subjected to endometrial biopsies. Samples were obtained with an endosampler (Pipelle, Cornier, Neully-en-Telle, France) at the time of surgery, as previously described [18]. Tissue was stored frozen at −80 °C until subsequent mRNA extraction, and RT procedures were achieved to obtain cDNA following routine methodology [19].

Approximately 2 µL containing 100 ng of equivalent cDNA from each endometrial biopsy were pipetted on the original array plates from OriGene. Once the plates had been set up, RANK isoforms were amplified by PCR and its relative expression normalized as described below so as to explore for the existence of quantitative and qualitative differences (i.e., alteration of the pattern of RANK isoforms) associated to the analyzed parameters of the endometrial tumors.

### 4.2. IHC Detection of RANK in TMA

Immunohistochemical staining of RANK was performed on arrayed tissue by using a human RANK/TNFRSF11A antibody (MAB6831—R&D Systems Inc., Minneapolis, MN, USA) coupled to a Dako (Glostrup, Denmark) REAL^©^ EnVision^©^ Peroxidase/DAB+, Rb/Mo Detection System (catalogue number K500711). Initial pilot experiments were conducted with samples of normal endometrium in order to optimize the concentration of RANK antibody and related IHC step procedures. In brief, sections were dewaxed by incubation in an oven at 60 °C for 60 min and rehydrated routinely. Antigen retrieval was performed by heating at 120 °C (under 1.9 bar pressure) for 2 min and submerging slides into Dako Low Retrieval Solution (i.e., citrate buffer at pH = 6). Endogenous peroxidase was quenched by incubation of the slide with 3% H_2_O_2_ in PBS at room temperature for 15 min. Subsequently, slides were incubated overnight at 4 °C with RANK primary antibody diluted 1:150 in Dako antibody diluent. On the next morning, the slides were washed with PBS and incubated for 20 min at RT with the HRP-link included in the Dako kit following manufacturer instructions. Detection of peroxidase was revealed using 3,3′-diaminobenzidene and subsequently the TMA was counterstained for 30 s with Harris haematoxylin, rehydrated, and mounted for visualization. Negative controls were included in each experiment by incubating tissue sections with antibody dilution buffer instead of the primary RANK antibody. Positive control slides consisted of triple-negative breast cancer and giant cell tumor of bone.

### 4.3. Quantification of IHC RANK Signalling in TMA

Spots in the array were photographed at different magnifications (10× or 20×) for descriptive and quantitative analysis of RANK signaling. Specifically, for quantification purposes, four random high-power fields (20×) were photographed per core/spot in the TMA with the use of an image analysis system linked to a Nikon Eclipse E400 microscope (Nikon, Tokyo, Japan). Due to the reduced size of each spot (1.5 mm), the number of images taken (4) was sufficient to embrace and represent the extension of the whole tissue in each core. The density of RANK staining was estimated as a function of RANK distribution and intensity signaling. For such purposes, the brown-stained (RANK) area of interest in each image was initially outlined and highlighted by using the segmentation tool included in Image Pro Plus 6.0 (Media Cybernetics Inc., Silver Spring, MD, USA) software analysis. Subsequently, the optical density of each individual pixel included in the outlined area of interest was obtained so that RANK density was expressed as the summation of the optical densities (Integrated Optical Density (IOD) parameter) provided by outlined pixels.

### 4.4. QF-PCR Amplification and RANK and Quantitative Analysis

OriGene endometrial cancer array supplemented plates were loaded into each well with 30 µL of a master mix stock solution containing 2× SYBR green mix (Life technologies, Carlsbad, CA, USA), water, and specific primers at 0.05 µm final concentration. The sequence of primers for β*-*actin, RANK (TNFRSF11A) and its variant RANK isoforms lacking exon 9 (*TNFRSF11A_Δ9*), exons 8–9 (*TNFRSF11A_Δ8,9*), and exons 7–9 (TNFRSF11A_Δ7,8,9), has been previously described [20]. Real-time PCR was performed using an ABI PRISM 7500 Sequence Detection System (Perkin Elmer Corp., Norwalk, CT, USA) according to the manufacturer instructions with a heated lid (105 °C), an initial denaturation step at 95 °C for 10 min, followed by 40 cycles of 95 °C for 15 s and 60 °C for 1 min. Relative expression level of RANK was calculated with the comparative 2^ΔΔ^*^C^*^t^ method, where Δ*C*_t_ = *C*_t(target)_ − *C*_t(control)_, ΔΔ*C*_t_ = *C*_t(target)_ − *C*_t(calibrator)_ and all samples were normalized to the *β-actin* gene.

In order to quantify alterations in profiling pattern of RANK isoforms associated with cancer parameters, the expression of each RANK isoform per case was normalized by dividing its specific 2^ΔΔ^*^C^*^t^ value by the summation of 2^ΔΔ^*^C^*^t^ values from the four RANK isoforms. Results were expressed as percentage.

### 4.5. Statistical Analysis

Statistical analysis was performed using SPSS 23.0, and the data were expressed as mean ± SD or standard error of the mean (SEM), where appropriate. For single comparisons (i.e., presence/absence of affected nodes or metastases and differences in RANK protein and mRNA expression between normal and pathological cases), a Student’s *t*-test or a nonparametric Mann–Whitney *U* test were employed. For multiple comparisons, cases were grouped according to age, histological grade, and tumor stage, and one-way ANOVA followed by a post hoc (Fisher’s LSD) analysis or a nonparametric Kruskal–Wallis followed by Mann–Whitney tests were employed to detect specific differences between groups. A bivariant Pearson or Spearman’s Rho correlation test for normally and nonnormally distributed samples were respectively employed to detect correlations between RANK expression and age of patients, histological grade, and tumor stage. Statistical significance was defined as *p* < 0.05.

## 5. Conclusions

We found that the IHC expression of RANK was higher in malignant than in normal endometrium and that RANK IHC score correlated with tumor grade. Gene expression, however, did not change between normal and malignant tissue. Future work should investigate whether the difference is reproduced in larger tumor series and, in such cases, which are the molecular determinants. Also, further work should clarify whether RANK expression relates to metastatic disease and survival.

## Figures and Tables

**Table 1:** Clinicopathological features of the 36 endometrial carcinoma cases investigated with immunohistochemistry.

Parameter	Variable	N	%
Age	<50	14	38.88
	≥50	22	61.12
Grade	I	4	11.11
	II	17	47.22
	III	13	36.11
	Unreported	2	5.56
Stage	I	23	63.89
	II	9	25.00
	III	3	8.33
	Unreported	1	2.78

**Table 2:** Clinicopathological features of the 40 endometrial carcinoma cases investigated with RT-QF-PCR in the tissue cDNA microarray.

Parameter	Variable	N	%
Age	<50	5	12.50
	≥50	35	87.50
Grade	I	2	5.00
	II	21	52.50
	III	17	42.50
Stage	I	12	30.00
	II	7	17.50
	III	13	32.50
	IV	8	20.00
M-Stage *	M1	7	17.50
	MX	33	82.50
N-Stage **	N0	19	47.50
	N1	11	27.50
	Nx	10	25.00

* N0, N1, NX respectively denote that nodes are negative, positive, or could not be assessed. ** M1, MX respectively denote that distant metastases are positive or could not be assessed.

## Supplementary Materials

Supplementary materials can be found at http://www.mdpi.com/1422-0067/19/7/1848/s1. Data supporting the results reported in this article are kept in an ad hoc created data-base stored in the Department of Pediatrics, Obstetrics and Gynecology of the School of Medicine, University of Valencia, Spain. Access to those data is available under request.
