# Supplementary figures and images for: Receptor Activator of Nuclear Factor Kappa B (RANK) and Clinicopathological Variables in Endometrial Cancer: A Study at Protein and Gene Level
Source: Int J Mol Sci. 2018 Jun 22;19(7):1848. doi: 10.3390/ijms19071848 (PMC6073139; doi:10.3390/ijms19071848)

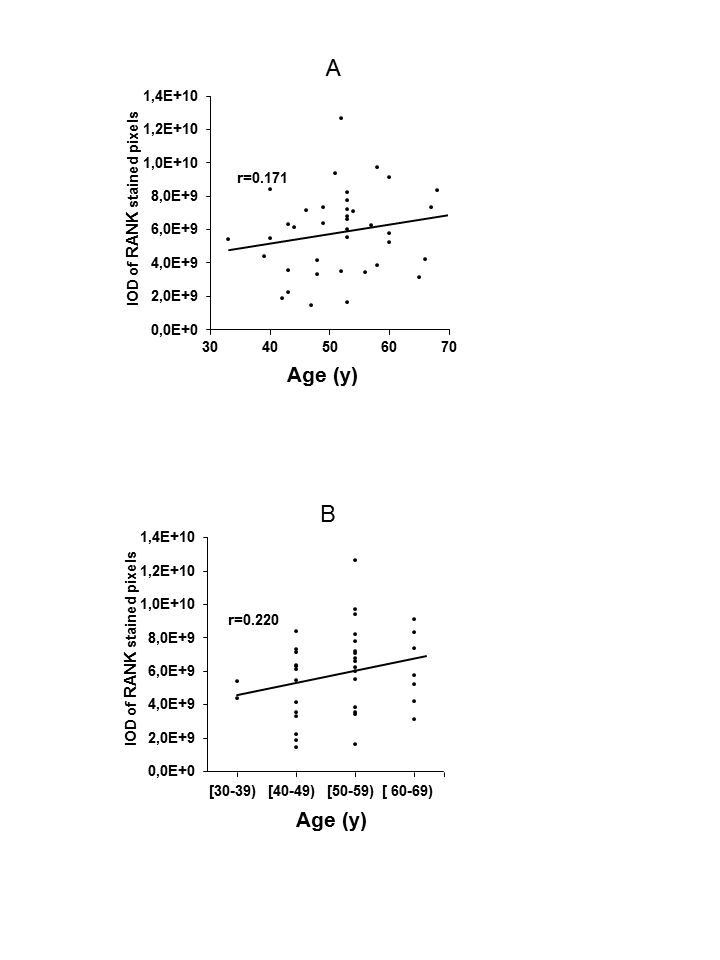

Supplement: Supplementary file 1 [file ijms-19-01848-s001.zip › S1.TIF]

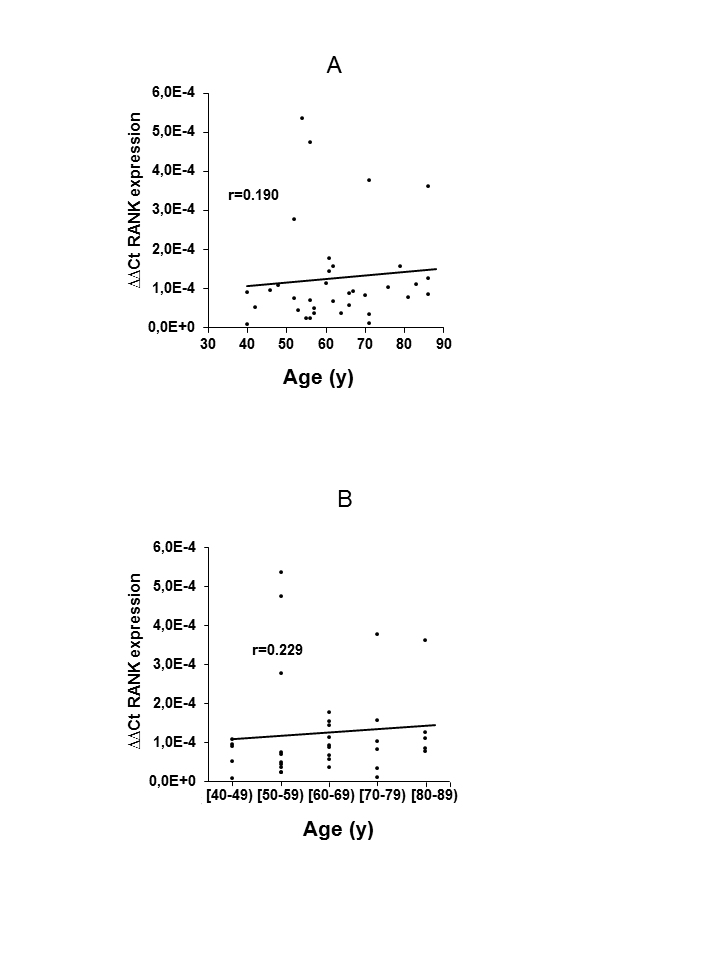

Supplement: Supplementary file 1 [file ijms-19-01848-s001.zip › S2.TIF]

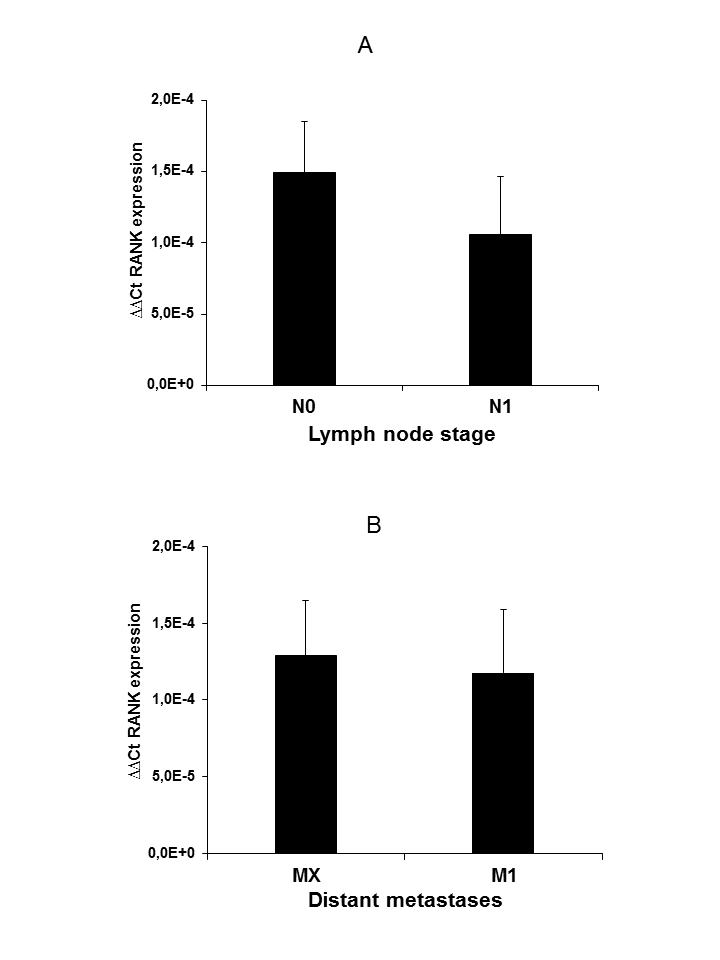

Supplement: Supplementary file 1 [file ijms-19-01848-s001.zip › S3.TIF]

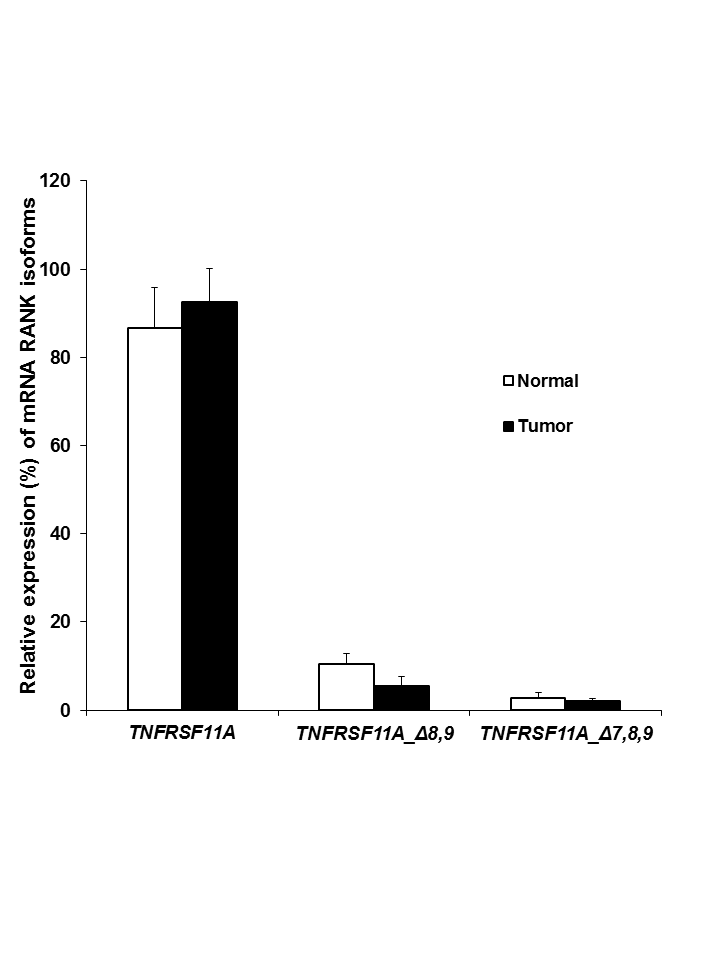

Supplement: Supplementary file 1 [file ijms-19-01848-s001.zip › S4.TIF]
